# Supplementary material for: Activity–Rest Circadian Rhythm of the Pearly Razorfish in Its Natural Habitat, before and during Its Mating
Source: Biology (Basel). 2023 Jun 2;12(6):810. doi: 10.3390/biology12060810 (PMC10295166; doi:10.3390/biology12060810)
Supplement: Supplementary file 1 [file biology-12-00810-s001.zip › biology-2301488-supplementary.pdf]

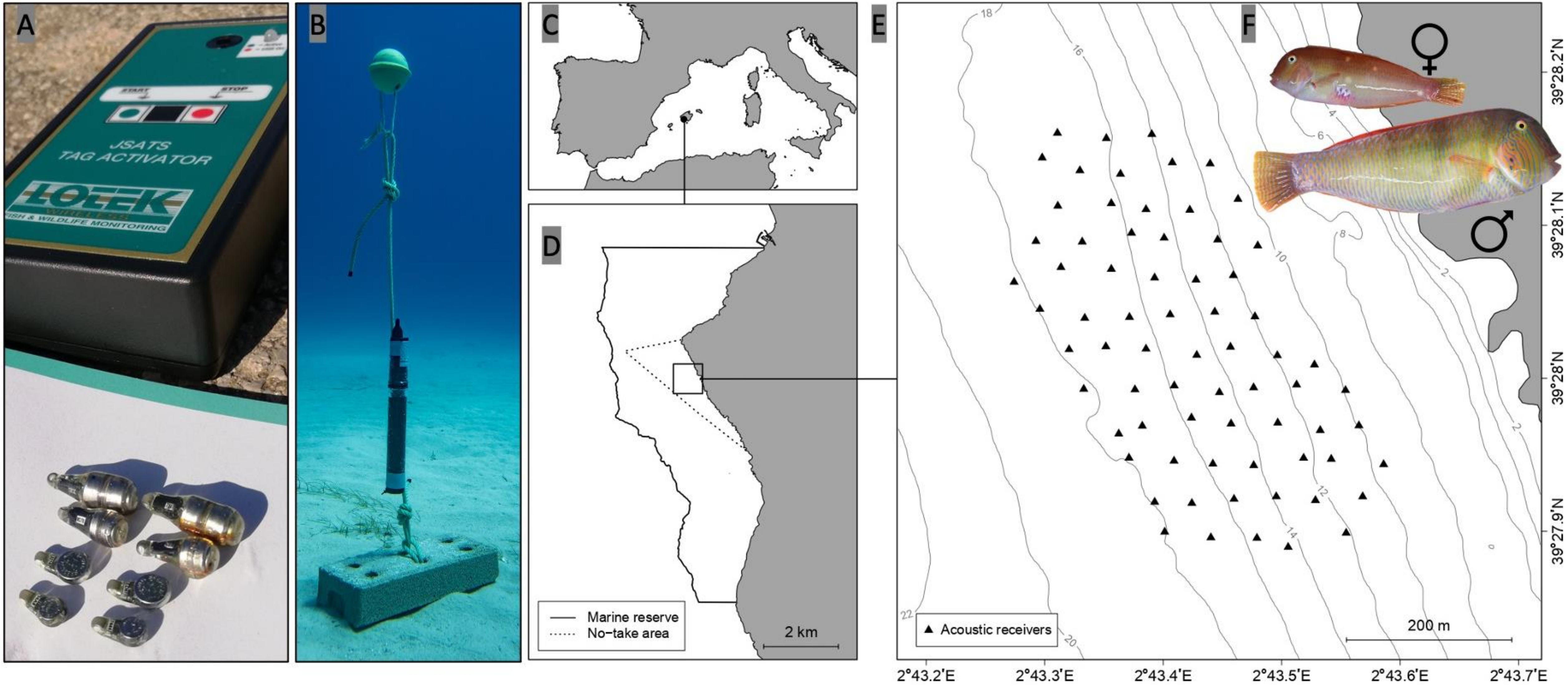

Figure S1. Supplementary Material. Details of the configuración of the high-resolution tracking system JSATS from "Lotek Wireless Inc.". (A) Image of different models of JSATS AMT transmitters that were impalnted to fish and the Activator of tags, (B) one of the 70 receivers model WHS-4250L deployed in the study-área, (C) location in the NW Mediterranean of the study-dite, (D) location of the study-site within the notake área of the Bay of Palma marine protected área, south of Mallorca island, € defenitive acoustic array of 70 units of WHS-4250L receivers deployed for this study, (F) and image of male and female of *Xyrichtys novacula*.
